# Stem cell factor’s role in enhancing the quality of fertilized and cloned porcine embryos for improved embryonic stem cell derivation

**DOI:** 10.3389/fvets.2023.1285530

**Published:** 2023-11-16

**Authors:** Lian Cai, Sang-Hwan Hyun, Eunhye Kim

**Affiliations:** ^1^Laboratory of Veterinary Embryology and Biotechnology (VETEMBIO), College of Veterinary Medicine, Chungbuk National University, Cheongju, Republic of Korea; ^2^Graduate School of Veterinary Biosecurity and Protection, Chungbuk National University, Cheongju, Republic of Korea; ^3^Institute for Stem Cell & Regenerative Medicine (ISCRM), Chungbuk National University, Cheongju, Republic of Korea; ^4^Laboratory of Molecular Diagnostics and Cell Biology, College of Veterinary Medicine, Gyeongsang National University, Jinju, Republic of Korea

**Keywords:** stem cell factor, *in vitro* fertilization, somatic cell nuclear transfer, embryonic stem cell, porcine

## Abstract

Stem cell factor (SCF), a cytokine growth factor, is expressed in various tissues of the male and female reproductive organs, including the testis, ovary, and endometrium. Its primary function involves cell survival, differentiation, and proliferation, achieved through its binding to the c-kit receptor. This study aimed to scrutinize the effects of SCF treatment during *in vitro* culture (IVC) on both the developmental potential and the efficiency of establishing embryonic stem cells (ESCs) from fertilized and cloned porcine embryos. The rates of cleavage and blastocyst formation exhibited no significant differences between fertilized and cloned embryos, even with the addition of SCF. However, it’s worth noting that embryos cloned with Cloud eGFP as donor cells demonstrated notably increased rates of hatched blastocysts when treated with SCF, and this increase was statistically significant (*p* < 0.05). Furthermore, following the complete dissection of the blastocysts, although there was no significant difference in the SCF-treated group, the area of expansion was significantly reduced (*p* < 0.01) in the group treated with the antagonistic blocker (ACK2) compared to both the control and SCF-treated groups. These outcomes suggest that the SCF/c-kit signaling pathway might play a pivotal role in embryo implantation. As anticipated, the efficiency of deriving ESCs was significantly higher (*p* < 0.01) in the group subjected to SCF treatment (12.82 ± 1.02%) compared to the control group (5.41 ± 2.25%). In conclusion, this study highlights the crucial role of SCF in enhancing the quality of porcine embryos, a vital step in obtaining high-quality ESCs.

## 1. Introduction

Due to their organ size, anatomical, and physiological similarities to humans, pigs have gained considerable attention as valuable large animal models in translational biomedical research (1). These models are utilized to study human diseases such as Alzheimer’s disease (2), cardiovascular diseases (3, 4), and diabetes mellitus (5). Pigs are also considered a promising source of organs for transplantation, either through xenotransplantation (6) or the creation of human-porcine interspecies chimeras (7). Especially, porcine pluripotent stem cells are conducive to the study of blastoid generation and the relevant molecular mechanism, which can be able to relatively free from ethical and legal constraints including the “14-day rule” (8, 9). The establishment of these models relies on generating porcine embryos *in vitro*, often involving gene modification through CRISPR/Cas9 microinjection into zygotes or transfection of donor cells (10). However, *in vitro*-produced porcine embryos face challenges like lower pregnancy rates and smaller litter sizes compared to their *in vivo* counterparts, which could be attributed to impaired embryo manipulation or gene editing. Both *in vitro* and *in vivo*, the initial pregnancy rate on day 25 can reach up to 80%. Most porcine fetal loss, occurring after day 25 and until day 45, is typically due to gradual fetal degeneration in the uterus, ultimately leading to disappearance (11).

To address these issues and establish pregnancy, effective bidirectional communication between the developing conceptus and the maternal reproductive tissue is essential (12). During this period, diffusible factors synthesized by the uterine endometrium are transferred to the fetus through the placenta, facilitating proper fetal development (13). Research suggests that stem cell factor (SCF), also known as Kit ligand (KITL), a multipotent cytokine, is expressed in various reproductive tissues and cell types including follicular cells, oocytes, spermatogenic cells, endometrial tissues, and embryos from the two-cell to hatched stages (14–19). SCF plays roles through its receptor, c-Kit, in paracrine and autocrine effects during germ cell development and embryo implantation. In mice, Taniguchi et al. found that co-culturing embryos with human granulosa cells or exposing them to human recombinant SCF improved hatching rates via the SCF/c-Kit system (20). Additionally, Lim et al. demonstrated that this system triggers blastomere cleavage and proliferation in mouse embryos through the *PI3K*-*Akt*-*tuberin*-*mTOR* cascade (20). In pigs, SCF is localized in endometrial stromal tissue in both pregnant and non-pregnant individuals (21). Jung et al. revealed that endometrium-secreted SCF induces the expression of phosphorylated AKT and its downstream target proteins, 70 kDa ribosomal S6 kinase (P70RSK), and ribosomal protein S6 (RPS6), in trophectoderm (Tr) cells derived from Day 12 conceptuses. SCF also accelerates the migration of Tr and uterine luminal epithelial cells, with these effects being inhibited by the *PI3K*/*Akt* inhibitor LY294002 (22). This suggests that SCF is present in the *in vivo* porcine environment and is essential for regulating conceptus development during the peri-implantation stage. However, the role of SCF in porcine preimplantation embryos and subsequent establishment of embryonic stem cells (ESCs) remains unclear.

This study delves into the effects of SCF supplementation during *in vitro* culture (IVC) on porcine preimplantation embryonic development potential and the efficiency of establishing ESCs. The rates and patterns of cleavage and blastocyst formation were assessed after *in vitro* fertilization (IVF) and somatic cell nuclear transfer (SCNT). To confirm the influence of the type of donor cells on the effectiveness of SCF, cloned embryos derived from two different types of transgenic cell lines were used. Furthermore, aspects like trophoblast spreading area, attachment rate, primary outgrowth numbers, and established cell line numbers were analyzed. To comprehend the necessity of SCF/c-Kit signaling during porcine IVC, a pharmacological inhibitor, ACK2, was utilized to specifically bind to and block c-Kit function.

## 2. Materials and methods

### 2.1. Reagents

Unless explicitly stated, all chemicals and reagents employed in this experiment were obtained from Sigma-Aldrich (St. Louis, MO, United States).

### 2.2. Oocyte collection and *in vitro* maturation

Porcine ovaries were procured from a local slaughterhouse and transferred to the laboratory in physiological saline within 2 h, maintaining a temperature range of 35–37°C. Using a 10 mL disposable syringe coupled with an 18-gauge needle, antral follicles (3–6 mm in diameter) were aspirated. The follicular fluid containing cumulus-oocyte complexes (COCs) was collected and allowed to settle for 10 min at 37°C in 15 mL conical tubes. Once sedimentation occurred, the mixture was resuspended in HEPES-buffered Tyrode’s medium with 0.05% (w/v) polyvinyl alcohol (PVA; TLH-PVA) after discarding the supernatant (23). Under a stereomicroscope (SMZ645; Nikon, Tokyo, Japan), COCs displaying uniformly granulated ooplasm were meticulously selected and subjected to three washes with TLH-PVA. Sixty COCs per group were individually cultured in separate wells of a 4-well dish (Nunc; Thermo Fisher Scientific, Waltham, MA, United States) containing 500 μL of tissue culture medium 199 (M199; Invitrogen, Carlsbad, CA, United States). This medium was supplemented with 10% (v/v) porcine follicular fluid, 0.6 mM cysteine, 10 ng/mL epidermal growth factor, 0.91 mM sodium pyruvate, 75 μg/mL kanamycin, 1 μg/mL insulin, and 10 IU/mL equine and human chorionic gonadotropins (from Daesung Microbiological Labs, Uiwang, Korea). The COCs were then incubated at 39°C in an atmosphere of 5% CO_2_ with humidified air for the initial 22 h, followed by transfer to a hormone-free medium for an additional 18–20 h. Following 42 h of maturation, oocytes were denuded through gentle pipetting with 0.1% hyaluronidase, followed by three washes with TLH-PVA. Oocytes exhibiting visible first polar bodies and homogeneous ooplasm were meticulously selected for subsequent embryo production.

### 2.3. *In vitro* fertilization

Weekly shipments of liquid semen were provided by Darby Genetics Inc. from the Department of Livestock Research in Anseong City, Gyeonggi-do, Republic of Korea. Upon receipt, the semen was temporarily stored at a temperature of 17°C. Subsequently, the semen underwent a double wash procedure using Dulbecco’s phosphate-buffered saline (dPBS; LB 001-02; WELGENE, Gyeongsan, Gyeongsangbuk-do, South Korea) with 0.1% bovine serum albumin (BSA). This washing process involved centrifugation at 2,000 × g for 2 min. The resulting sperm pellet was then resuspended in a modified Tris-buffered medium (mTBM) (24). It is important to note that the mTBM had been pre-equilibrated for a minimum of 8 h at 39°C in an environment consisting of 5% CO_2_ and 95% air. To ensure the suitability of the sperm for fertilization, a pre-fertilization evaluation of sperm motility was conducted, and only samples containing over 80% motile sperm were used for subsequent experiments.

For the fertilization process, a 5 μL sperm suspension was introduced into a 40 μL droplet of mTBM. This droplet housed 15 mature porcine oocytes, and suitable dilution was performed to achieve a final sperm concentration of 5 × 10^5^ sperm/mL. Following a 20 min co-incubation period, loosely attached sperm cells were delicately removed from the zona pellucida of the oocytes using precise pipetting techniques. After undergoing an additional double wash, these oocytes were incubated in fresh mTBM for a period of 5 to 6 h. The entire fertilization procedure was performed within a controlled environment at a temperature of 39°C within a humidified environment comprising 5% CO_2_ and 95% air.

### 2.4. Donor cell culture

Prior to performing somatic cell nuclear transfer (SCNT), two genetically modified cell lines were utilized: cloud eGFP fetal fibroblasts (C-eGFP-FF) and Jeju #3-SBone-Dppb-pigTYR-CreERT2 (Braf). These cell lines were thawed and cultivated in Dulbecco’s modified Eagle’s medium (DMEM; Gibco, Carlsbad, CA, United States) enriched with high levels of glucose. This medium was supplemented with 10% fetal bovine serum (FBS; Gibco, Carlsbad, CA, United States), 1× nonessential amino acids (NEAA; Gibco, Carlsbad, CA, United States), 1× glutamine (Gibco, Carlsbad, CA, United States), 0.1 mM β-mercaptoethanol (Gibco, Carlsbad, CA, United States), and 1× Antibiotic-Antimycotic (Gibco, Carlsbad, CA, United States), all of which served as growth factors for the cells. To synchronize the cells in the G0/G1 phase of the cell cycle, they were cultivated until they reached confluence. For the SCNT procedure, only cells that had undergone less than 10 passages were used.

### 2.5. Somatic cell nuclear transfer

To visualize the nuclei, fully matured oocytes were incubated for 5 min in a calcium-free TLH solution with 0.2% BSA (TLH-BSA), containing 5 μg/mL Hoechst 33342 (B-2265), and 5 μg/mL cytochalasin B (CB, C6762). After undergoing two washes, the polar bodies and ooplasm containing metaphase II chromosomes within the oocytes were gently aspirated using a 16 μm glass pipette (Origio Humagen Pipets, Charlottesville, VA, United States), submerged in a droplet of TLH-BSA supplemented with 5 μg/mL CB. Enucleation success was confirmed by momentary ultraviolet (UV) exposure, facilitated by a shutter system (VCM-D1; Vincent Associates, Rochester, NY, United States).

Following this step, a trypsinized donor cell (Diameter: 14–15 μm), characterized by a smooth cell surface and showing green fluorescence under UV irradiation, was delicately inserted into the perivitelline space of the enucleated oocyte using a fine injecting pipette. The coupled cells were subjected to two washes in a 280 mM mannitol solution (pH: 7.0–7.4; Osmolarity: 280 mOsm/L) containing 0.001 mM CaCl_2_ and 0.05 mM MgCl_2_, before being positioned between two electrodes of a 1 mm chamber, which were covered with a 260 mM mannitol solution (pH: 7.0–7.4; Osmolarity: 260 mOsm/L) containing 0.1 mM CaCl_2_ and 0.05 mM MgSO_4_. The activation and fusion process for these coupled cells were carried out using two direct current pulses of 160 V/mm for a duration of 60 μs.

After three washes, successful membrane fusion was confirmed by observing the fused oocytes under a stereomicroscope after 30 min of incubation in TLH-BSA. Following this confirmation, the fused oocytes were placed in porcine zygote medium-3 (PZM-3) containing 2 mM 6-dimethylaminopurine and 0.4 mg/mL demecolcine. This incubation occurred over a period of 4 h at 39°C within a humidified atmosphere of 5% CO_2_, 5% O_2_, and 90% N_2_.

### 2.6. Cultivation of embryos and assessment of developmental competence

After undergoing a thorough washing process, ten presumptive zygotes were individually cultured in micro-drops of PZM-3, each with a capacity of 25 μL. These micro-drops were subsequently covered with pre-warmed mineral oil, and the zygotes were cultured at a temperature of 39°C within a humidified environment comprising 5% O_2_, 5% CO_2_, and 90% N_2_. The initiation of IVF or SCNT marked the beginning as day 0. By day 2, the cleaved embryos were observed utilizing a stereomicroscope. Embryos demonstrating uniform cleavage were categorized into three developmental stages based on the number of blastomeres: 2–3, 4–5, and 6–8 cells. These embryos were subsequently transferred to fresh PZM-3 medium for continued cultivation. On day 7, the blastocysts were meticulously evaluated and categorized according to their extent of expansion and hatching conditions, following the classification methods detailed in our prior study (25). This categorization encompassed early blastocysts (characterized by a compact blastocyst with a blastocoel size equal to or less than half of the embryo volume), expanded blastocysts (defined as a sizable blastocyst with a blastocoel larger than half of the embryo volume, or a blastocyst where the blastocoel fully occupied the embryo), and hatched blastocysts (referring to a blastocyst either in the process of hatching or one that has already hatched).

### 2.7. Preparation of feeder cells

The feeder cell layer for culturing ESCs was established using mouse fetuses obtained from the Institute of Cancer Research on embryonic day 13.5. After excising the fetal heads, internal organs, and legs, the remaining tissues were finely minced in dPBS and subjected to two washes through centrifugation (2000 rpm for 3 min). Subsequently, the isolated mouse embryonic fibroblasts (MEFs) were cultured in the aforementioned growth medium and incubated at 37°C in an atmosphere of 5% CO_2_ and air. To render the MEFs inactive, a concentration of 10 μg/mL mitomycin C (Roche, Basel, Switzerland) was introduced to passage 2–3 MEFs for a duration of 2–2.5 h. The inactivated MEFs were then seeded at a density of 5 × 10^5^ cells/mL in a 4-well dish coated with 0.5% gelatin (EMD Millipore) in the growth medium.

### 2.8. Establishment and culture of ESCs lines

The zona pellucida was removed from blastocysts using a 0.5% protease solution. After undergoing three washes and microscopic inspection to ensure integrity, fully intact blastocysts were placed onto pre-prepared feeder cells in ESCs medium. The medium composition was as follows: low glucose DMEM/F10 (Gibco, Carlsbad, CA, United States) containing 1× glutamine, 1× NEAA, 1× Antibiotic-Antimycotic, 0.1 mM β-mercaptoethanol, 4 ng/mL basic fibroblast growth factor (bFGF, Invitrogen Corporation, Carlsbad, CA, United States), and 15% FBS. Following 2 days of cultivation, the attachment efficiency was gauged by calculating the ratio of attached colonies to the total number of initially plated blastocysts. Blastocysts that remained stationary when the dish was gently swirled were categorized as attached. On the 9th day, the count of primary outgrowths was determined. For the purpose of passaging, colonies reaching 3–4 mm in size were isolated and fragmented into several clumps using glass pipettes. After a brief wash with ESCs medium, these colony clumps were reseeded onto fresh, inactivated feeder cells. Feeder cells were replenished with new ESCs medium at least 2 h prior to passaging. Subculture was conducted mechanically every 5–9 days under enzyme-free conditions. Meanwhile, daily medium replacement was carried out, and all cell cultures were incubated at a temperature of 37°C with 5% CO_2_ under humidified conditions.

### 2.9. Evaluation of trophoblast spreading

At 48 h after plating, we assessed the degree of trophoblast spreading through phase-contrast microscopy. To quantify the area of trophoblast spreading, we measured both the horizontal (*a* axis) and longitudinal (*b* axis) diameters for each attached blastocyst colony. This analysis was conducted using ImageJ software^1^ and was further guided by the equation of an ellipse: Area = *π* × *a* × *b*/4.

### 2.10. Detection of alkaline phosphatase activity

The established presumptive ESC lines were subjected to three washes with dPBS and subsequently fixed in a 4% paraformaldehyde solution for 10 min at a temperature of 25°C. The NBT/BCIP chromogen stock solution (Roche, Basel, Switzerland) was diluted using Tris solution (0.1 M Tris, NaCl, pH 9.48), and this mixture was added to the wells containing the ESCs. Following an incubation period at room temperature lasting 20 to 60 min, and subsequently, three washes with Tris solution, the activity of ALP was visualized under a light microscope and captured using a Leica camera.

### 2.11. Experimental design

**Figure 1:**
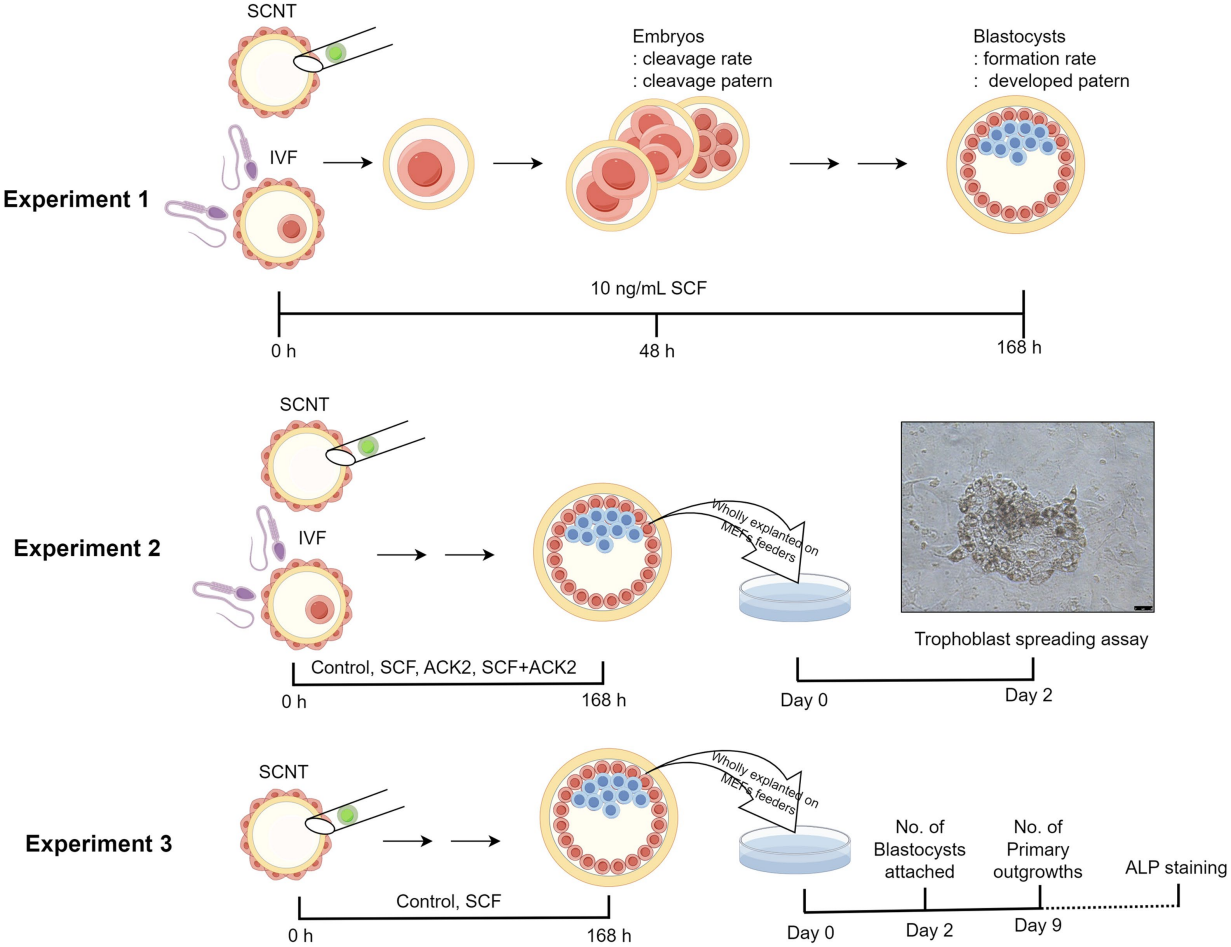
Illustration depicting the experimental design. Three experiments were planned in total. In experiment 1, the effects of SCF supplementation during IVC on the developmental potential of porcine embryos was investigated. In experiment 2, the interplay between SCF and c-Kit on the implantation capacity of blastocysts was examined. Finally, in experiment 3, the effects of SCF on the establishment efficiency of porcine ESC lines was evaluated. IVF, *in vitro* fertilization; SCNT, somatic cell nuclear transfer; SCF, stem cell factor; ALP staining, Alkaline phosphatase staining. This figure was generated using Figdraw 2.0 (www.figdraw.com) and the export ID is TWRUI11554.

As shown in Figure 1, a total of three experiments were designed. In Experiment 1, we investigated the effects of SCF supplementation during IVC on the developmental potential of porcine embryos. Based on our previous study (19), 10 ng/mL SCF was treated, followed by the assessment included cleavage rate, cleavage pattern, blastocyst formation rate, and blastocyst pattern after IVF or SCNT. To confirm the impact of the origin of donor cells on the effects of SCF, cloned embryos were produced from two different types of cell lines (C-eGFP-FF and Braf) and evaluated separately. In Experiment 2, we examined the interplay between SCF and c-Kit on the implantation capacity of blastocysts. The spreading surface area of trophoblasts was measured after implanting whole blastocysts under feeder cells. During IVC, 10 μg/mL of ACK2 (eBioscience, San Diego, CA, United States), an SCF antagonist, was introduced. Experiment 3 focused on analyzing the effects of SCF on the derivation efficiency of porcine ESC lines. This phase involved evaluating the count of attached blastocysts and primary outgrowths. Additionally, we established stable cell lines capable of undergoing three consecutive passages.

### 2.12. Statistical analysis

Each experiment was conducted with a minimum of three replicates. Statistical analyses were carried out using SPSS Statistics (version 21.0; SPSS Inc., Chicago, IL, United States). Data comparisons were executed using either Student’s *t*-test or one-way analysis of variance (ANOVA) as deemed suitable. Additionally, one-way ANOVA was employed with Duncan’s multiple range tests. Specific details regarding the statistical methodologies, corresponding *p*-values, and replication specifics for each experiment can be found in the legends of the respective tables or figures. Data were expressed as the mean ± standard error of the mean (SEM). Statistical significance was determined as *p* < 0.05, unless stated otherwise.

## 3. Results

### 3.1. Effects of SCF supplementation during IVC on the developmental capacity of porcine embryos after IVF

**Figure 2:**
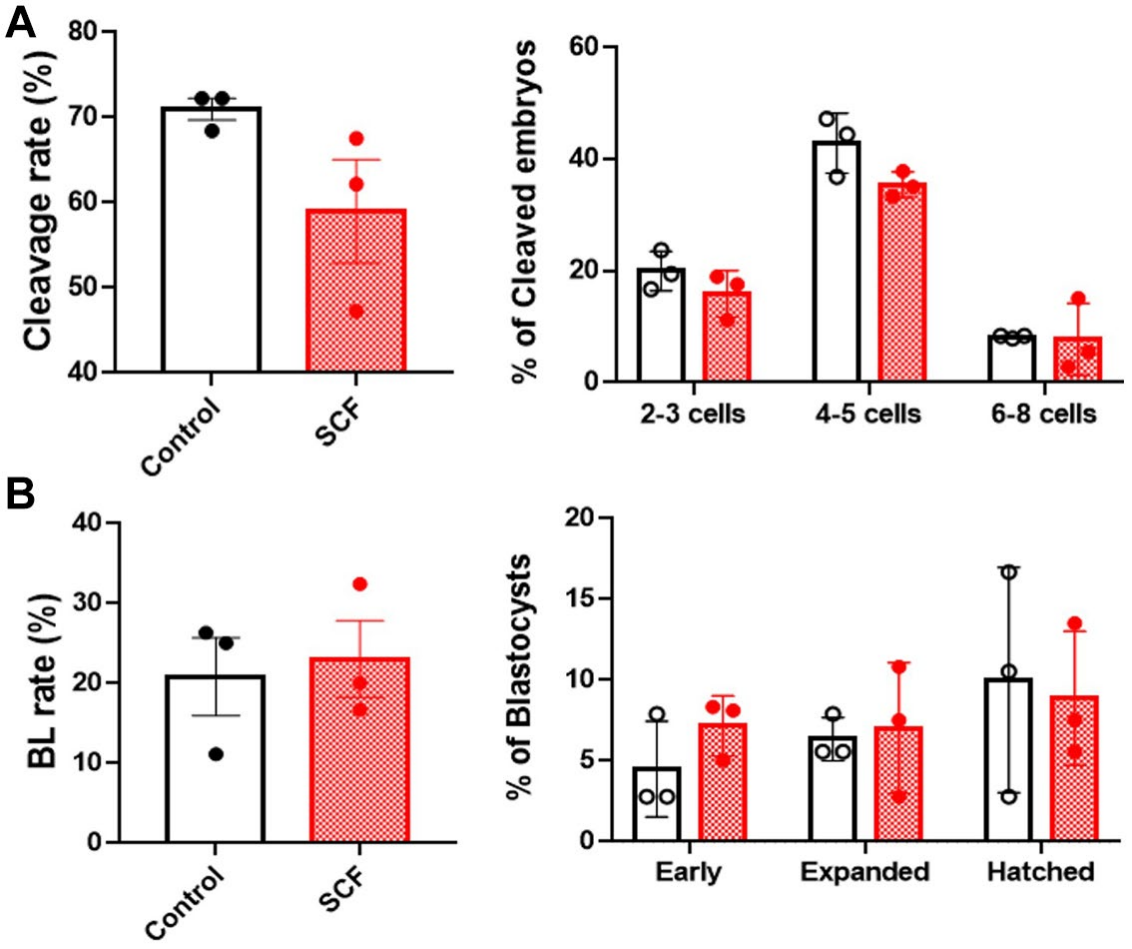
Production of *in vitro* fertilized (IVF) porcine blastocysts using SCF treatment during *in vitro* culture (IVC). **(A)** Cleavage rate and pattern (2–3, 4–5, and 6–8 cell stage embryos) of embryos on day 2 after IVF. **(B)** Blastocyst formation rates and patterns (early, expanded, and hatched stage embryos) on day 7 after IVF. BL, blastocysts; SCF, 10 ng/mL stem cell factor. Statistical significance was assessed using Student’s *t*-test. Data are presented as mean ± SEM.

*In vitro* fertilized porcine embryos were generated with or without supplementation of 10 ng/mL SCF during IVC. Two days post-fertilization, cleavage rates and patterns were assessed (Figure 2A). After 7 days, blastocyst formation rates and patterns were examined (Figure 2B). Nonetheless, as depicted in Figure 2, there were no significant differences observed in the developmental potential of porcine embryos in the SCF-treated group compared to the control.

### 3.2. Effects of SCF supplementation during IVC on the developmental capacity of porcine embryos after SCNT

**Figure 3:**
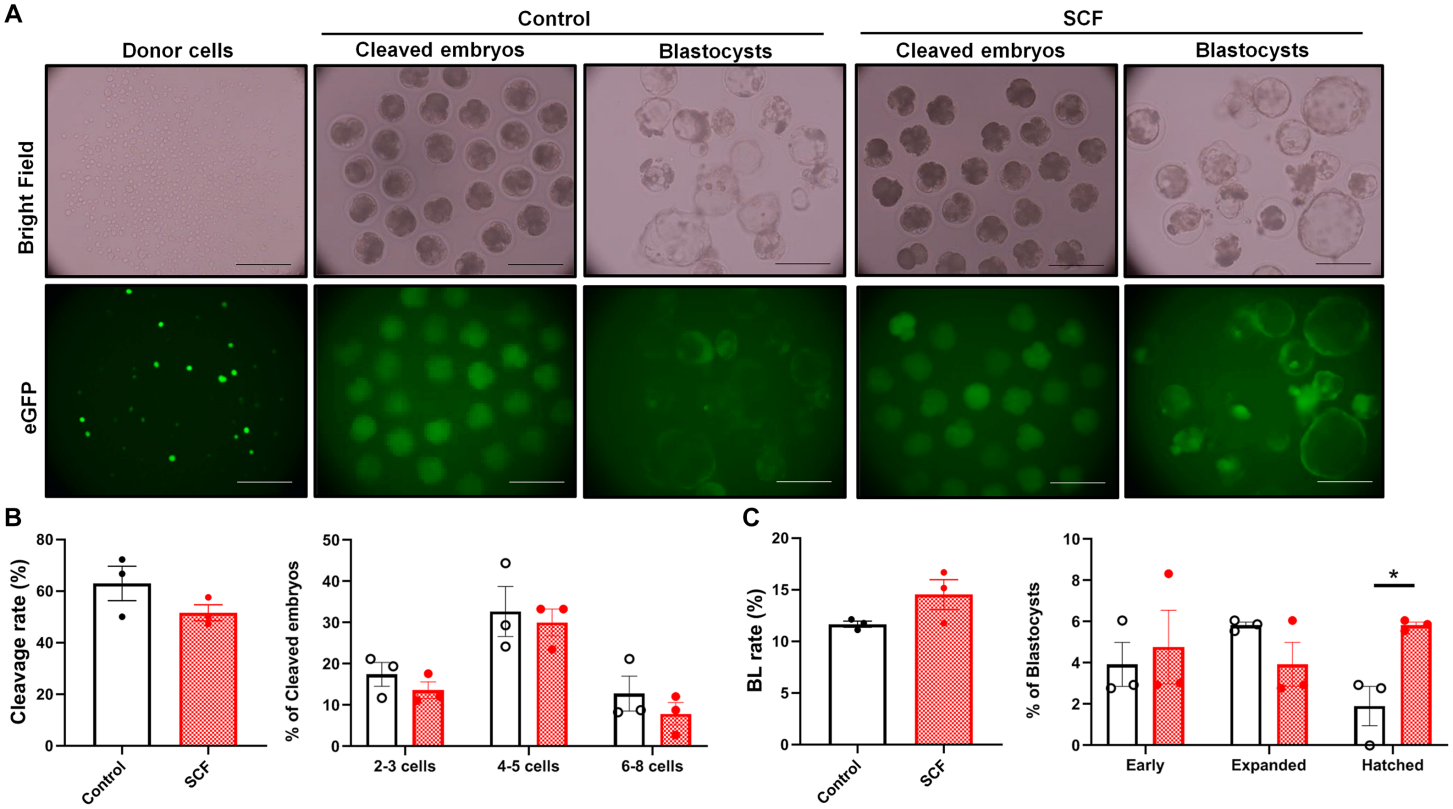
Production of cloned porcine blastocysts with Cloud eGFP fetal fibroblasts as donors using SCF treatment during *in vitro* culture (IVC). **(A)** Representative bright field and fluorescent (green) images of single donor cells, cleaved embryos (day 2), and blastocysts (day 7). Scale bars = 200 μm. **(B)** Cleavage rates and patterns (2–3, 4–5, and 6–8 cell stage embryos) of embryos on day 2 after somatic cell nuclear transfer (SCNT). **(C)** Blastocyst formation rates and patterns (early, expanded, and hatched stage embryos) on day 7 after SCNT. Statistical significance was determined using Student’s *t*-test. Data are presented as mean ± SEM. SCF: 10 ng/mL stem cell factor. *p < 0.05.

**Figure 4:**
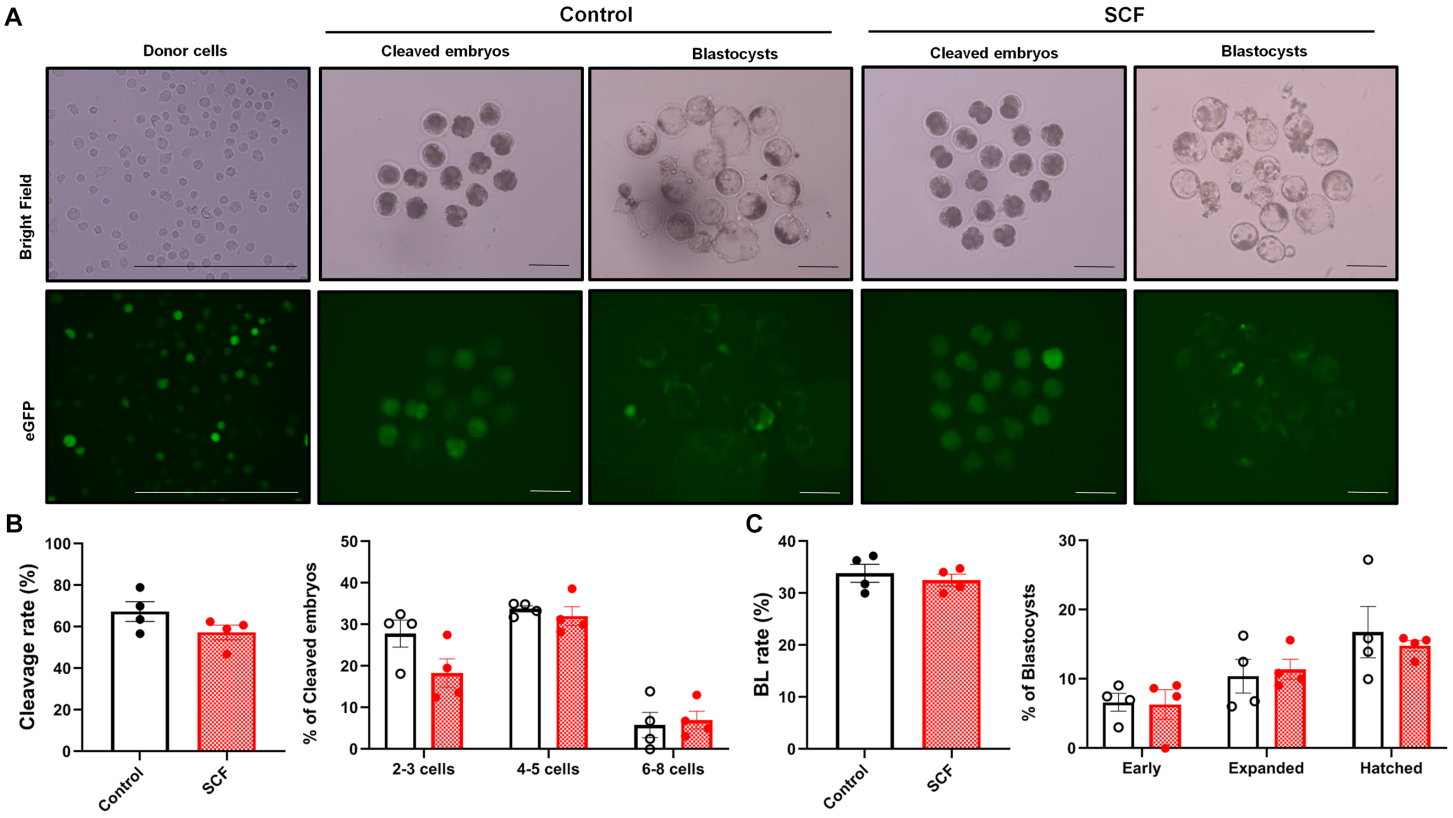
Production of cloned porcine blastocysts with Jeju #3-SBone-Dppb-pigTYR-CreERT2 (Braf) as donor cells using SCF treatment during *in vitro* culture (IVC). **(A)** Representative images of bright field and fluorescent (green) of single donor cells, cleaved embryos (day 2), and blastocysts (day 7). Scale bars = 200 μm. **(B)** Cleavage rates and patterns (2–3, 4–5, and 6–8 cell stage embryos) of embryos on day 2 after somatic cell nuclear transfer (SCNT). **(C)** Blastocyst formation rates and patterns (early, expanded, and hatched stage embryos) on day 7 after SCNT. Statistical significance was determined using student’s *t*-test. Data are presented as mean ± SEM. SCF: 10 ng/mL stem cell factor. *p < 0.05.

To explore the impact of SCF treatment during IVC on embryonic developmental competence after SCNT, cloned porcine embryos were generated with or without 10 ng/mL SCF supplementation during IVC. Meanwhile, these embryos were produced from two different donor cell origins (C-eGFP-FF and Braf) and were verified separately. In this result, embryos originating from Braf (approximately 30%) exhibit almost three times higher blastocyst formation rates than those derived from C-eGFP-FF (approximately 10%) (Figures 3, 4). Whereas, intriguingly, C-eGFP-FF-derived embryos showed a significantly (*p* < 0.05) higher rate of hatched blastocysts in the SCF-treated group than in the control (Figure 3C), but not Braf-derived embryos (Figure 4C). Nevertheless, no differences in cleavage rates, cleavage patterns, and blastocyst formation rates were observed between the SCF-treated and control groups, not only the embryos derived from C-eGFP-FF but also from Braf (Figures 3, 4).

### 3.3. Effects of SCF supplementation during IVC on trophoblast spreading area after complete exfoliation of IVF- and SCNT-derived blastocysts

**Figure 5:**
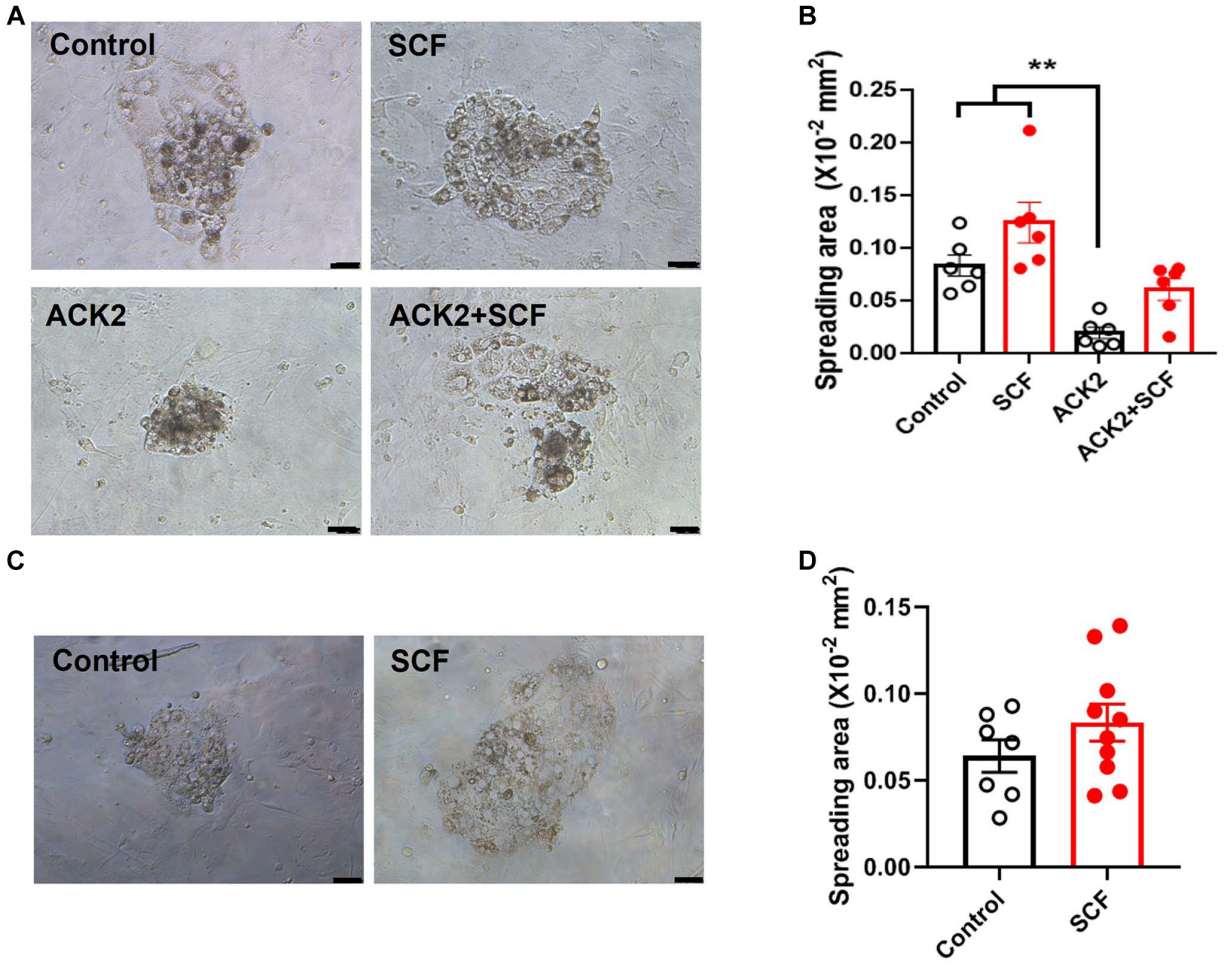
Quantification of trophoblast spreading area on day 2 after wholly explanting the *in vitro* fertilized (IVF) and cloned blastocysts. (**A, C**) Representative morphology of attached blastocysts derived from IVF **(A)** and somatic cell nuclear transfer (SCNT), using Jeju #3-SBone-Dppb-pigTYR-CreERT2 (Braf) as donor cells **(C)**, on day 2 after seeding. Porcine embryos were cultured with or without 10 ng/mL SCF and its antagonistic blocker (ACK2), respectively. After 7 days of culture, blastocysts were wholly planted on mouse embryonic fibroblasts (MEFs) feeders. Scale bars = 200 μm. (**B, D**) Comparison of trophoblast spreading area between various groups (control, SCF, ACK2, and ACK2 + SCF) on day 2 after seeding the IVF **(B)** and cloned **(D)** blastocysts. Statistical significance was determined using one-way ANOVA in panel (B) and Student’s *t*-test in panel **(D)**. Data are presented as mean ± SEM. SCF: 10 ng/mL stem cell factor. **p < 0.01.

To further assess the impact of SCF treatment during IVC on stem cell establishment, we measured the trophoblast spreading area on the second day after seeding IVF and cloned embryos. For SCNT-derived blastocysts, we attempted to verify using those from both types of donor cell origins. Unfortunately, unlike Braf-derived embryos, the blastocyst formation rate of that from C-eGFP-FF origin is too low resulting in great limitation in analyzing (Data not shown). Additionally, to evaluate the effect of SCF/c-Kit signaling inhibition, ACK2, an antagonistic blocker of c-Kit, was employed during IVC. Thereby, porcine embryos were cultured in an IVC medium supplemented with SCF, ACK2, and SCF + ACK2. The result showed that there are no significant differences (*p* < 0.05) in the SCF-treated groups of IVF (Figures 5A,B) or cloned blastocysts (Figures 5C,D) compared to the control. However, the ACK2-treated groups displayed a significantly decreased (*p* < 0.05) trophoblast spreading area 48 h after seeding compared to the control and SCF-treated group (Figures 5A,B).

### 3.4. Effects of SCF supplementation during IVC on the efficiency of porcine embryonic stem cell establishment

**Figure 6:**
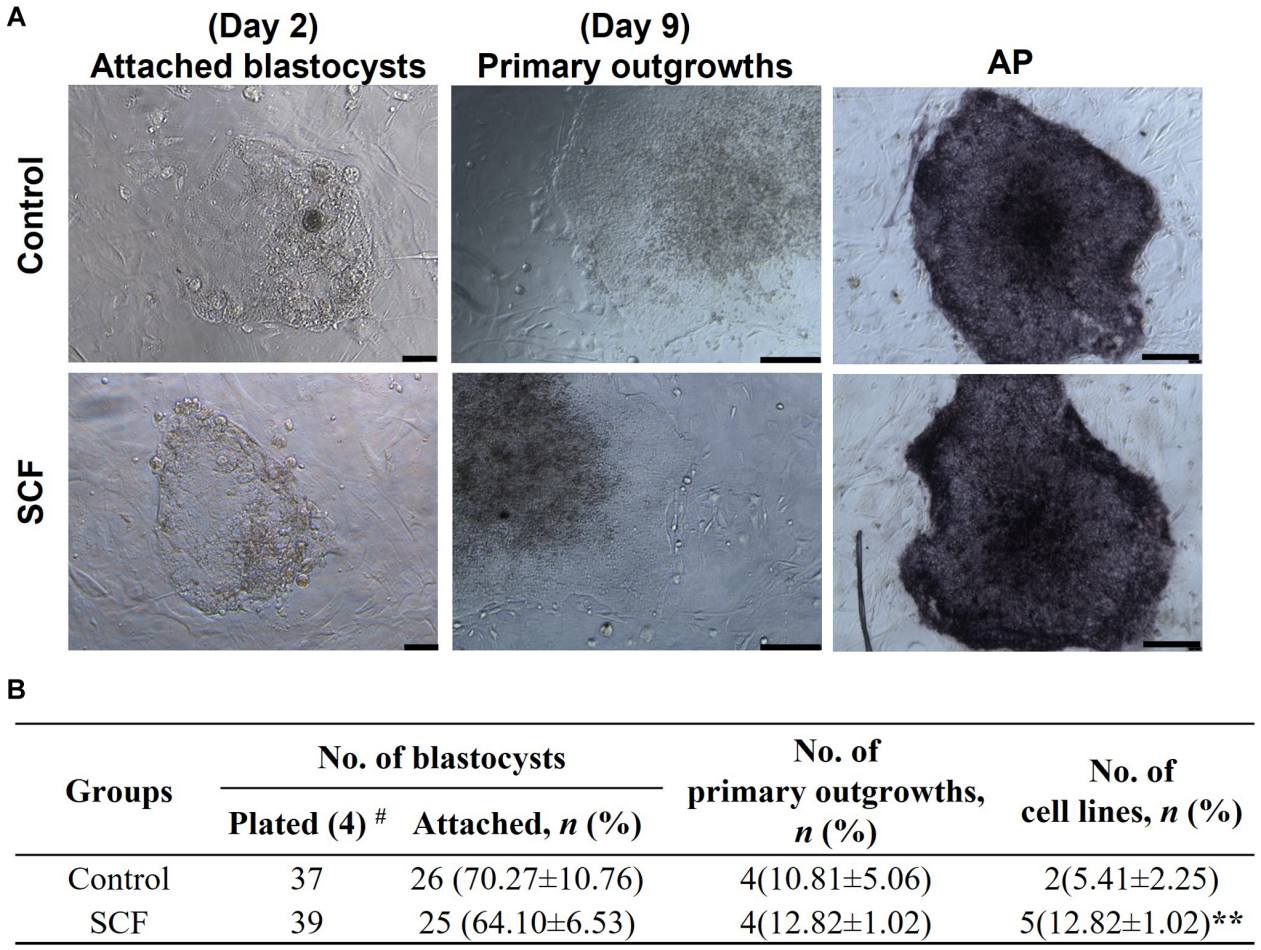
Establishment efficiency of porcine embryonic stem cells (ESCs) using Jeju #3-SBone-Dppb-pigTYR-CreERT2 (Braf)-derived cloned blastocysts after culturing with stem cell factor (SCF). **(A)** Representative morphology of attached blastocysts (day 2) and primary outgrowths (day 9) after seeding on mouse embryonic fibroblasts (MEFs) feeders, sub-cultured colonies (passage 2), and alkaline phosphatase (AP) activity of established stem cell lines. Scale bars = 200 μm. **(B)** Porcine ESCs derivation efficiency using cloned blastocysts cultured with or without 10 ng/mL SCF *in vitro*. Statistical significance was determined using Student’s *t*-test. Data are presented as mean ± SEM. SCF: 10 ng/mL stem cell factor. **p < 0.01 and #Replication number.

After cultivation with or without 10 ng/mL SCF supplementation, cloned blastocysts were entirely placed onto the MEF feeder cells on the sixth day. A total of 37 and 39 blastocysts were plated in the control and SCF-treated groups, respectively. The attachment rate, number of primary outgrowths, and efficiency of establishing ESC lines were evaluated. As shown in Figure 6A, each porcine ESC line from SCNT blastocysts exhibited morphological changes during derivation. Two days post-seeding, small round cells were encircled by giant cells originating from the trophectoderm. Following 9 days of culture, the primary outgrowths demonstrated similar morphological changes in both the control and SCF-treated groups. Primary colonies were mechanically detached and fragmented into clumps using pulled glass pipettes. In porcine ESC medium, both subcultured colonies displayed the characteristic morphology of porcine ESCs (passage 2), including the formation of a tightly packed epithelial cell-like morphologies with distinct boundaries and strong alkaline phosphatase activity. Throughout ESC derivation, no significant differences were noted in the attachment and outgrowth rates of porcine ESC lines between the SCF-treated and control (Figure 6B). However, the efficiency of establishing a porcine ESC line in the SCF-treated group was significantly higher than that in the control group (Figure 6B). These outcomes suggest that porcine ESC lines can be effectively derived from porcine NT in SCF-supplemented medium.

## 4. Discussion

In this study, we have demonstrated that SCF treatment did not significantly impact the developmental competence of embryos derived from IVF and SCNT. Nevertheless, the number of embryos reaching the hatched stage increased, and the efficiency of establishing ESC lines was augmented in cloned blastocysts. Additionally, following seeding, ACK2 (an anti-c-Kit antibody) reduced the trophoblast spreading area, which was subsequently restored by SCF, indicating the crucial role of SCF/c-Kit signaling in successful porcine embryo implantation and appropriate maternal-fetal communication.

Embryos *in vivo* typically enter the uterine cavity, encountering uterine gland-secreted fluid rich in complex bioactive molecules such as amino acids, proteins, ions, carbohydrates, and lipids. This milieu supports precise blastocyst development and crucial endometrium–embryo interactions (26, 27). Unfortunately, embryos cultured *in vitro* lack this maternal microenvironment, leading to reduced quality and viability. To address this, researchers have explored various maternally secreted factors, including the colony-stimulating factor family (28, 29), epidermal growth factor (30), and prostaglandin F2α (31), to enhance *in vitro* culture systems for porcine embryos. SCF is widely recognized for its pleiotropic roles, including cell proliferation, differentiation, apoptosis, and migration, mediated by its receptor, c-Kit, across diverse cell types (32, 33). Recent studies indicate that c-Kit is expressed in human and mouse embryos and uterine endometrium, and its interaction with SCF promotes pre-implantation embryonic development by regulating downstream targets (15, 20, 34, 35). Moreover, in murine embryos, exogenous SCF supplementation improves blastocyst formation and blastomere proliferation, which is compromised by c-Kit gene silencing (20). Encouragingly, SCF mRNA is transcribed in the porcine uterine cycle, with elevated levels during early pregnancy. Additionally, SCF induces c-kit expression in the trophectoderm and uterine endometrium, underlining the critical role of the SCF/c-kit mechanism in porcine conceptus development and implantation (22). Nonetheless, understanding of SCF’s function in porcine preimplantation embryonic development and implantation potential is limited, motivating the current correlated research.

In contrast to mouse results, our findings did not show significant effects on the cleaved embryo rate and blastocyst formation rate not only in IVF but also in SCNT embryos of pigs, following SCF supplementation. However, the rate of blastocysts progressing to the hatched stage consistently increased in cloned embryos using the C-eGFP-FF cell line as donor cells. Hatched blastocysts are known to possess higher potential for implantation and pregnancy post-transfer (36), likely due to their relatively increased number of trophectoderm cells directly interacting with the maternal endometrium (37). This suggests that exogenous SCF could enhance the quality of porcine embryos, thereby promoting their implantation. However, this effect was not replicated in embryos cloned using Braf as donors. This discrepancy likely arises from differences in cell types (Yucatan and Jeju domestic pigs) or gene editing, as previous studies have shown that the genomic reprogramming status during nuclear transfer varies based on these factors, determining which genes are activated and thereby affecting clone fetal development efficiency (38). Indeed, our results demonstrated a relatively higher blastocyst formation rate in cloned embryos from the Braf cell line (approximately 30%) compared to C-eGFP-FF (10–15%). SCF showed more pronounced effects on embryos with donor cells of lower developmental potential.

Finally, we investigated the effects of SCF on the implantation potential of porcine blastocysts, especially focused research on the establishment efficiency of cloned ESCs, since this model has great application value in the study of human reproductive disease via generating patient-derived blastoid or gastruloids (8, 9), but its derivation efficiency is still low, unlike fertilized ESCs (39, 40). Trophoblastic cells, key adhesive cells that transform into trophoblastic giant cells localized at the maternal-fetal interface, possess a spreading area that can be considered a marker of implantation potential (15). We investigated the trophoblast outgrowth’s spreading area to affirm SCF’s impact on the implantation potential of porcine blastocysts. Similar to results in mice (15, 20), the surface area tended to increase in blastocysts cultured with SCF. Conversely, blastocysts nearly lost their spreading potential with the anti-c-Kit antibody ACK2, and SCF restored this inhibitory effect. In our seeding system, wholly planted blastocysts maintained their expanded structure until attachment to suitable substrates, exhibiting higher attachment and spreading potential than shrunken blastocysts. Furthermore, porcine blastocysts tend to shrink after approximately seven days in culture, especially those of poor quality. This suggests that, akin to mice, the c-Kit protein may be present in porcine blastocysts, and SCF-c-Kit interaction is vital for pre- and peri-implantation processes in porcine embryos.

In fact, due to limited total cell numbers and poor quality, cloned blastocysts face greater challenges in establishing ESC lines compared to *in vitro* fertilized or parthenogenetically activated blastocysts (40). Despite numerous attempts by researchers over the past decade, the efficiency of establishment in pigs has remained in the low single-digit percentage range (41–43). In this study, we present, for the first time, evidence that the derivation efficiency of porcine-cloned ESC lines was increased by SCF, reaching an enhancement of nearly two-fold. This suggests that SCF/c-Kit signaling may be essential for porcine ESCs’ self-renewal and maintenance of stemness. In the current study, the specific molecular regulatory mechanism of SCF has not been elucidated yet. To clarify this, the characterization of cloned ESCs lines, and transcriptomic analysis need to be conducted further. Despite these limitations, our study provides preliminary insights into the translational biomedical research of cloned ESCs in the porcine species as a large animal-human disease model.

## 5. Conclusion

In summary, our findings provide evidence that the interaction between SCF and c-Kit is pivotal for pre-implantation embryonic development and the establishment of ESC lines in pigs. Moreover, we believe that adding SCF to porcine embryonic culture media can significantly enhance the efficiency and cost-effectiveness of this research model.

## Data availability statement

The original contributions presented in the study are included in the article/supplementary material, further inquiries can be directed to the corresponding authors.

## Ethics statement

The animal study was approved by the Ethics Committee for Animal Experimentation at Chungbuk National University (permit number CBNUA-1460-20-02). The study was conducted in accordance with the local legislation and institutional requirements.

## Author contributions

LC: Data curation, Formal analysis, Investigation, Methodology, Visualization, Writing – original draft, Writing – review & editing. S-HH: Conceptualization, Funding acquisition, Project administration, Supervision, Validation, Writing – review & editing. EK: Conceptualization, Funding acquisition, Investigation, Methodology, Project administration, Supervision, Validation, Writing – original draft, Writing – review & editing.

## Footnotes

^1^ https://imagej.net/Downloads
